# Hepatitis D virus infection in Isfahan, central Iran: Prevalence and risk factors among chronic HBV infection cases

**Published:** 2011-04-01

**Authors:** Behrooz Ataei, Mohammad Reza Yazdani, Hamid Kalantari, Majid Yaran, Zary Nokhodian, Abbas Ali Javadi, Anahita Babak, Peyman Adibi

**Affiliations:** 1 Isfahan Infectious Diseases Research Center, Isfahan University of Medical Sciences, Isfahan, IR Iran; 2 Isfahan Liver Diseases Research Center, Isfahan University of Medical Sciences, Isfahan, IR Iran; 3 Community and Preventive Medicine Specialist, Infectious Diseases and Tropical Medicine Research Center, Isfahan University of Medical Sciences, Isfahan, IR Iran

**Keywords:** Hepatitis B infection, Hepatitis D infection, Prevalence, Iran

## Abstract

**Background:**

Hepatitis D virus (HDV) is dependent on hepatitis B virus (HBV) infection. Acute infection with HDV can occur simultaneously with acute HBV infection or be superimposed onto a chronic HBV infection.

**Objectives:**

This study aimed to identify cases of HDV and determine its prevalence in patients with chronic HBV infection for the first time study in Isfahan, central Iran.

**Patients and Methods:**

In a cross-sectional study in 2009, 346 who had been diagnosed for at least 6 months with chronic HBV were enrolled consecutively. Anti-HDV was measured by ELISA in the serum of these patients.

**Results:**

The study included 245 males (70.8%) and 101 (29.2%) females with a mean age of 39 ± 12.4 years. Anti-HDV was present in 8 (3.5%) HBe antibody-positive patients (p = 0.36) and in 2 (2.3%) HBe antigen-positive cases (p = 0.68). No association was found between hepatitis D and probable risk factors.

**Conclusions:**

This study demonstrates that the prevalence of HDV infection is higher in patients who are positive for HBeAb compared those who are HBeAg-positive. Therefore, most HDV antibody-positive cases in Isfahan are HBV/HDV superinfections but not coinfections.

## Background

Hepatitis B (HBV) is one of the most common public health problems worldwide, especially in developing countries, causing 1 million deaths per year [1]. According to available estimates, 1.2% to 9.7% of the world's population and approximately 2.14% of the Iranian population have an HBV infection [2]. Such patients not only are a source of infection but also are susceptible to late complications of this virus, such as chronic active hepatitis, cirrhosis, and liver cancer [3].

Hepatitis D virus (HDV) is a defective RNA virus that depends on the hepatitis B surface antigen (HBsAg) of hepatitis B virus for its replication, developing exclusively in patients with acute or chronic hepatitis B. Simultaneous infection with HDV tends to accelerate the progression of chronic HBV infection to chronic active hepatitis, cirrhosis, and hepatocellular carcinoma and mediates fulminant hepatitis. In addition, the response of HDV patients to antiviral therapy and the required dosages of therapeutic regimens differ from those of chronic hepatitis B alone [4][5].

Approximately 5% of patients with chronic hepatitis B infection worldwide are infected with hepatitis D virus. Its prevalence in Italy, eastern Europe, and western Asia is higher than in the rest of the world, reaching 83.3%, 8.3%, and 12.5% in Romania, Italy, and Russia, respectively [5][6][7].

HDV has a broad geographical distribution with two dominant patterns of transmission. In endemic regions, such as southern Italy, parts of Africa, and South America, it is transferred through close personal contact in the absence of clear skin contact, such as close personal relationships among members of a family. In contrast, in areas that have a low prevalence, such as western Europe and North America, HDV is seen more commonly in groups with frequent skin contact such as continual recipients of blood and blood products and intravenous drug users [8][9]. Sex and maternal-child transmission are other modes of transmission [1][4].

The most frequent method of diagnosis of HDV infection is measurement of anti-HDV (IgM, IgG) in serum by ELISA. PCR can also be used to detect viral RNA in blood [10][11].

Acute HDV infection can occur simultaneously with acute HBV infection or can be superimposed onto chronic HBV infection. Fatal fulminant hepatitis occurs in 20% to 30% of coinfections of HDV and HBV in humans versus 2% of patients with acute hepatitis B without HDV coinfection [1]. More than 3% of the Iranian population is infected with HBV, and few studies have been conducted to determine the prevalence of HDV in chronic HBV patients in Iran. No study has examined this topic in Isfahan, the second largest city in Iran.

## Objectives

This study was designed and performed in 2009 to detect anti-HDV in chronic HBV patients and related risk factors in this region.

## Patients And Methods

This cross-sectional study was performed from April to November 2009. We included patients with chronic hepatitis B virus infection who were in a carrier state (normal ALT, normal sonography, normal physical exam, and negative for HBeAg) or exhibited immune tolerance (normal ALT, normal sonography, normal physical exam, and HBeAg-positive) and under surveillance at the Infectious Diseases and Tropical Medicine Research Center, Isfahan University of Medical Sciences, with at least 6 months of diagnosis. In these patients, HBsAg was detected by individual, familial, or disease-related follow-up, and they were tested periodically to measure viral activity and liver function.

Sampling was performed by nonprobability purposive technique. Consecutive patients of all ages and both genders were included in the study, and patients with thalassemia and frequent recipients of blood and blood products were excluded. Persons who did not consent to participation in the study were not enrolled.

For all patients, demographics and risk factors were recorded and 5-mL blood samples were collected. Total HDV antibody was detected by ELISA (Diapro, Italy). Results on HBsAb, HBeAg, HBeAb, and HBV DNA were extracted from the patients' records. Laboratory results on other variables, such as age, sex, duration of hepatitis, methods of diagnosis, and risk factors of hepatitis, were recorded for each patient. Data were analyzed using SPSS, version 15, using descriptive statistical methods and chi-square, Fisher's exact, independent t, and logistic regression tests. P-values of less than 0.05 were considered significant.

## Results

Overall, 346 cases, comprising 245 men (70.8%) and 101 women (29.2%) with a mean age of 39 ± 12.4 years (40.4 ± 11.8 and 35.8 ± 13.2 years, respectively, p-value = 0.003), were studied. The median time from diagnosis of infection with hepatitis B virus was 3 years (range, 1 month to 31 years). With regard to marital status, 54 cases (15.6%) were single, 290 cases (83.8%) were married, and 2 patients (0.6%) were widowed.

With regard to mode of diagnosis, the HBV was detected by disease-related follow up in 130 cases (37.6%), familial check-up in 105 cases (30.3%), by individual testing in 30 cases (8.7%), after blood donation in 69 cases (19.9 %), during pregnancy in 10 patients (2.9%), and by congenital assay in 2 cases (0.6%).

The distribution of frequencies of risk factors for hepatitis B is shown in Table 1. Ten cases (2.9%) had antibodies against hepatitis D virus (6 males = 2.4% and 4 females = 4%, p-value = 0.44). The status of HDV infection relative to hepatitis B is shown in Table 2. We observed that 3.5% of HBeAb-positive patients and 2.3% of HBeAg-positive patients had HDV antibody; ie, the prevalence of HDV Ab was higher in HBeAb-positive patients compared with HBeAg-positive patients.

On analysis of demographic and risk factors by logistic regression, no statistically significant relationship was noted between hepatitis D and probable risk factors. We calculated odds ratios (ORs) with 95% confidence intervals (CI) of various factors, presented in Table 3. Multivariate logistic regression analysis was used to control for the confounding effects of other variables and estimate the adjusted odds ratios, shown in Table 3.

**Table 1:** Frequency of risk factors of hepatitis B virus infection in participants

**Variable**	**Number**	**Percentage**
**Intravenous drug users**	20	5.8
**Surgery**	144	41.6
**Blood transfusion**	40	11.6
**Transplantation**	1	0.3
**History of hepatitis in the family**	186	53.8
**Uterine curettage**	12	11.9^a^
**Dialysis**	13	3.8
**Dental manipulation**	292	84.4
**Tattoo**	54	15.6
**Ear Piercing**	91	26.3
**Circumcision**	243	99.2^b^
**High-risk sex**	6	1.7
**Phlebotomy**	60	17.3

^a^ only in females

^b^ only in males

**Table 2:** Frequency of hepatitis B markers relative to HDV antibody

** Marker **	** HDV antibody **		** p-value **
	** Positive **[No. (%)]	** Negative **[No. (%)]	
HBe antigen			
Positive	2 (2.3%)	86 (97.7%)	0.68
Negative	8 (3.1%)	248 (96.9%)	
HBe antibody			
Positive	8 (3.5%)	221 (96.5%)	0.36
Negative	2 (1.7%)	113 (98.3%)	
HBs antibody			
Positive	_	26 (100%)	_
Negative	_	16 (100%)	
HBV DNA			
Positive	1 (2.6%)	38 (97.4%)	0.23
Negative	2 (11.1%)	16 (88.9%)	

**Table 3:** Odds ratios of risk factors of hepatitis D virus infection

**Variable**	**OR** (95% CI)	**adjusted OR** (95% CI)
**Age**	0.97 (0.92-1.01)	0.96 (0.91-1.02)
**Sex **a	0.6 (0.16-2.19)	1.97 (0.42-9.23)
**Marital status **b	31.11 (1.79-537.93) e	1.12 (0.11-11)
**Duration of HBV infection**	1.02 (0.84-1.24)	1.03 (0.8-1.32)
**History of surgery **c	0.94 (0.26-3.41)	1.54 (0.36-6.6)
**History of blood transfusion **c	0.89 (0.11-7.24)	1.09 (0.12-10)
**History of hepatitis in the family **c	1.31 (0.36-4.74)	0.82 (0.21-3.14)
**History of dental manipulation **c	073 (0.15-3.57)	1.47 (0.29-7.5)
**History of Phlebotomy**	1.19 (0.24-5.74)	0.86 (0.15-4.75)
**HBe antigen **d	0.72 (0.15-3.46)	0.44 (0.02-8.04)
**HBe antibody **d	2.04 (0.42-9.79)	0.32 (0.02-5. 85)

^a^ female = 0 male = 1 (Reference category)

^b^ single and widowed = 0, married = 1 (Reference category)

^c^ has not had = 0, has had = 1 (Reference category)

^d^ negative = 0, positive = 1 (Reference category)

^e^ statistically significant

## Discussion

The prevalence of serum HDV antibody in our study group was 2.9%. In this study, a family history of hepatitis, phlebotomy, surgery, blood transfusions, and dental manipulations were the most frequent risk factors in patients with HDV antibody.

Based on studies in Mediterranean countries, including the Middle East, HDV infection is transmitted primarily through noncutaneous routes, especially close personal contact, such as that between family members [3]. Based on the results of this study and other reports, HDV transmission and spread can be prevented by avoidance of an infected person in close family relationships, and the disease can be diagnosed earlier by screening high-risk individuals and their families.

In 1990, Rezvan et al. detected HDV antibodies in 2.5% of asymptomatic HsAg carriers in Tehran, the capital of Iran [12]. Karimi et al. in 2000 reported a 1.3% prevalence of HDV in chronic carriers of hepatitis B in Tehran [13]. Amini et al. reported prevalence (2.4%) of HDV infection in similar research population in 1993 in Hamadan, western Iran [14]. In 2000 in Babol, northern Iran, Hassanjani-Roshan and Taheri observed HDV positivity in 2% of HBV carriers [3]. Roshandel and colleagues reported a 5.8% prevalence of HDV in Golestan, northern Iran, in 2008 [15]. In Tabriz, northwestern Iran, in 2002,,Torabi and colleagues noted a prevalence of HDV antibody of 0.6% in HBsAg-positive individuals [16]. Alavian et al. reported 5.7% HDV seropositivity among HBV-infected subjects in Iran [17]. These studies demonstrate that the prevalence of HBV/HDV coinfection and superinfection has increased in the past decade in Iran, during which the prevalence of acute and chronic hepatitis D has decreased worldwide [18].

The prevalence of HDV among HBsAg-positive individuals has been reported to be 1.5% in Yugoslavia [19], 1.6% in Spain [10], 2.2% in Taiwan [20], 4% in Mexico [21], 16.6% in Pakistan [22], 24.4% in Bangladesh [1], 12.5% in Russia [7], 83.3% in Romania [5], 23.6% in Japan [23], and 8.3% in Italy [6]. These studies suggest that the prevalence of HDV differs in various parts of the world and is higher in eastern Europe and western Asia. Our findings are consistent with the results of Hassanjani-Roshan, in which the rate of HDV infection in northern Iran was not significantly different between various age groups [3]. Moreover, this prevalence was slightly, but insignificantly, higher in women.

The prevalence of HDV antibody in HbeAg-positive patients was higher than in HBeAb-positive cases, but in our study, the prevalence of HDV antibody in HBeAb-positive patients was higher compared with HBeAg-positive persons. In Celen and colleagues, a significant relationship was reported in 2005 in Turkey between the duration of positivity of HBsAg and HDV antibody, but a significant relationship did not exist between HBeAg or antibody and HDV antibody [24]. We did not observe an association between the duration of HBV and HDV infection. Local data have shown that the progression of liver damage in cases of HBV infection are not low, suggesting that prognostic indicators, including coinfections and superinfections, must be determined [25].

In conclusion, researchers should be aware of the risk of coinfection and superinfection of HBV and HDV in this region. The prevalence of HDV in high-risk individuals in Iran has only been reported by Karimi and colleagues, who noted HDV antibody in 25.2% of dialysis patients in Tehran [13]. Therefore, it is necessary to assess the frequency of HDV in high-risk individuals, including intravenous drug users and continual recipients of blood and blood products, such as patients with thalassemia, hemophilia, and coagulation factor deficiencies, in Isfahan and other regions of Iran. In addition, studies are necessary to identify transmission routes of HDV in Isfahan and other regions of Iran to design programs for the prevention of HDV infection in the community.

Because liver lesions are more severe in patients with HBV/HDV coinfections and because the frequency of HDV in patients with liver lesions is unknown in Iran, it is necessary to determine the prevalence of HDV in patients with chronic active hepatitis, cirrhosis, liver cancer, and resistant chronic hepatitis B and in asymptomatic chronic carriers of hepatitis B in Isfahan and other parts of Iran separately.
